# Undifferentiated embryonic stem cells express ionotropic glutamate receptor mRNAs

**DOI:** 10.3389/fncel.2013.00241

**Published:** 2013-12-03

**Authors:** Svenja Pachernegg, Illah Joshi, Elke Muth-Köhne, Steffen Pahl, Yvonne Münster, Jan Terhag, Michael Karus, Markus Werner, Zhan-Lu Ma-Högemeier, Christoph Körber, Thomas Grunwald, Andreas Faissner, Stefan Wiese, Michael Hollmann

**Affiliations:** ^1^Department of Biochemistry I - Receptor Biochemistry, Ruhr University BochumBochum, Germany; ^2^International Graduate School of Neuroscience, Ruhr University BochumBochum, Germany; ^3^Ruhr University Research School, Ruhr University BochumBochum, Germany; ^4^DFG Graduate School 736, Ruhr University BochumBochum, Germany; ^5^Department of Cell Morphology and Molecular Neurobiology, Ruhr University BochumBochum, Germany; ^6^Department of Molecular and Medical Virology, Ruhr University BochumBochum, Germany; ^7^Group for Molecular Cell Biology, Department of Cell Morphology and Molecular Neurobiology, Ruhr University BochumBochum, Germany

**Keywords:** ESCs, neuroepithelial cells, neural stem cells, synaptic markers, qRT-PCR, patch-clamp recordings

## Abstract

Ionotropic glutamate receptors (iGluRs) do not only mediate the majority of excitatory neurotransmission in the vertebrate CNS, but also modulate pre- and postnatal neurogenesis. Most of the studies on the developmental role of iGluRs are performed on neural progenitors and neural stem cells (NSCs). We took a step back in our study by examining the role of iGluRs in the earliest possible cell type, embryonic stem cells (ESCs), by looking at the mRNA expression of the major iGluR subfamilies in undifferentiated mouse ESCs. For that, we used two distinct murine ES cell lines, 46C ESCs and J1 ESCs. Regarding 46C ESCs, we found transcripts of kainate receptors (KARs) (GluK2 to GluK5), AMPA receptors (AMPARs) (GluA1, GluA3, and GluA4), and NMDA receptors (NMDARs) (GluN1, and GluN2A to GluN2D). Analysis of 46C-derived cells of later developmental stages, namely neuroepithelial precursor cells (NEPs) and NSCs, revealed that the mRNA expression of KARs is significantly upregulated in NEPs and, subsequently, downregulated in NSCs. However, we could not detect any protein expression of any of the KAR subunits present on the mRNA level either in ESCs, NEPs, or NSCs. Regarding AMPARs and NMDARs, GluN2A is weakly expressed at the protein level only in NSCs. Matching our findings for iGluRs, all three cell types were found to weakly express pre- and postsynaptic markers of glutamatergic synapses only at the mRNA level. Finally, we performed patch-clamp recordings of 46C ESCs and could not detect any current upon iGluR agonist application. Similar to 46C ESCs, J1 ESCs express KARs (GluK2 to GluK5), AMPARs (GluA3), and NMDARs (GluN1, and GluN2A to GluN2D) at the mRNA level, but these transcripts are not translated into receptor proteins either. Thus, we conclude that ESCs do not contain functional iGluRs, although they do express an almost complete set of iGluR subunit mRNAs.

## Introduction

Excitatory neurotransmission in the vertebrate CNS is mainly mediated by ionotropic glutamate receptors (iGluRs), which can be divided into three main subfamilies: NMDA receptors (NMDARs), AMPA receptors (AMPARs), and kainate receptors (KARs) (Hollmann and Heinemann, 1994). Besides their function in synaptic signal transduction, iGluRs are involved in neural development by modulating the proliferation, migration, and differentiation of neural progenitors both during embryonic and adult neurogenesis (Cameron et al., 1995; Haydar et al., 2000; Deisseroth et al., 2004; Luk and Sadikot, 2004; Manent et al., 2005). For instance, iGluR activation in embryonic neocortical slices has been reported to lead to reduced DNA synthesis (Loturco et al., 1995) as well as increased proliferation (Haydar et al., 2000). Furthermore, it has been shown that iGluR activation increases the proliferation of neural progenitor cells (Luk and Sadikot, 2004; Brazel et al., 2005). However, this effect in different brain regions is mediated by different subclasses of iGluRs. While AMPAR and KAR activation promotes proliferation in cortical progenitors, NMDAR activation increases proliferation of striatal progenitors (Luk and Sadikot, 2004).

Whereas the impact of iGluRs on the proliferation and differentiation of neural progenitor cells and neural stem cells (NSCs) has been studied extensively (Schlett, 2006), their expression and putative function in undifferentiated embryonic stem cells (ESCs) is far less well understood. So far, it has only been demonstrated that neuron-like cells derived from ESCs show kainate- and NMDA-induced current responses (Bain et al., 1995; Finley et al., 1996; Kim et al., 2009), but the expression of potentially functional iGluRs in undifferentiated ESCs remained elusive.

Thus, we investigated the expression of functional iGluRs in undifferentiated ESCs by patch-clamp recordings and by iGluR expression analysis at the mRNA and protein levels in two distinct murine ESC lines. First, we used the well-defined ES cell line 46C, which expresses EGFP under the control of the Sox1 promoter (Ying et al., 2003; Conti et al., 2005; Muth-Kohne et al., 2010a,b). Since Sox1 is an early neuroectodermal marker, the generation of neuroepithelial precursor cells (NEPs) from 46C ESCs can easily be monitored via the expression of EGFP (Ying et al., 2003; Conti et al., 2005). NEPs can then be further differentiated into radial glia-like NSCs; and both NEPs and NSCs can be differentiated into neurons and glia, thus demonstrating their NSC character (Ying and Smith, 2003; Conti et al., 2005; Muth-Kohne et al., 2010a,b).

Regarding the expression of iGluRs in ESCs, we found subunits of all three major subfamilies of iGluRs to be expressed in ESCs at the mRNA but not at the protein level. Moreover, most of the iGluR subunits are not expressed at the protein level in 46C-derived NEPs or NSCs either. Additionally, we analyzed the expression patterns of pre- and postsynaptic markers in 46C ESCs, NEPs, and NSCs. Similar to our findings for iGluRs, all three cell types express glutamatergic synapse markers on the mRNA but not at the protein level. Lastly, patch-clamp recordings of 46C ESCs showed no current responses to direct AMPAR and KAR agonist application.

Additionally, we also checked the expression of iGluRs at the mRNA and protein levels in a different, independent ES cell line, namely J1 ESCs. We found transcripts of AMPAR, KAR, and NMDAR subunits to be expressed in undifferentiated J1 ESCs, but, similar to 46C ESCs, none of the investigated subunits was translated into receptor protein as confirmed by Western blotting.

Thus, we conclude the absence of functional iGluRs in undifferentiated ESCs.

## Materials and methods

### Cell culture

All cultured cells were maintained at 37°C and 5% CO_2_. The genetically engineered 46C ESC line (kind gift of Dr. Austin Smith) obtained from E14Tg2a.IV mouse ESCs (Aubert et al., 2003; Ying et al., 2003) was grown in GMEM containing 10% FCS, 10% tryptose phosphate, 0.1 mM 2-mercaptoethanole, 1.8 mM glutamine, and 1000 U/ml leukemia inhibitory factor (LIF) (Millipore). NEPs were differentiated from 46C ESCs in a neuroinductive medium as described previously (Ying and Smith, 2003). NSCs were generated from NEPs by the prolonged cultivation in N2B27 medium. The proliferative state of NSCs was maintained by adding EGF and FGF-2 (both 10 ng/ml; Peprotech) to the N2B27 medium (Conti et al., 2005). J1 ESCs were grown in R1 medium (DMEM containing 20% FCS, 0.05 mM 2-mercaptoethanole, 2.0 mM glutamine, 1x non-essential amino acids, and 1000 U/ml LIF).

### Reverse transcription and quantitative real time PCR

Total RNA was isolated from tissue and cultured cells using the GeneElute Mammalian Total RNA Miniprep Kit (Sigma). Two microgram of total RNA of each sample were used for reverse transcription (SuperScript II Reverse Transcriptase; Invitrogen). For quantitative Real Time PCRs (qRT-PCRs), 50 ng cDNA per reaction was used as template. qRT-PCRs were performed on a Roche LightCycler (Roche) using the LightCycler Fast Start DNA Master Plus SYBR Green I Kit (Roche) according to the manufacturer's manual. The reaction was performed with an initial pre-incubation for 10 min at 95°C, followed by 40 amplification cycles (95°C for 10 s, 59°C for 10 s, and 72°C for 20 s). A melting cycle was performed to determine melting temperatures of the amplified products (95°C for 0 s, 65°C for 15 s, and melting by increasing the temperature to 95°C at a rate of 0.1°C/s). Whole brain RNA of neonatal (P3) C57BL/6 mice served as positive control. For primer sequences, see Table 1.

**Table 1:** **List of primers used in the quantitative RT-PCRs**.

**Gene**	**Primer sequence (shown in 5′ → **3**′)**
GluA1 (s)	Gacaactcaagcgtccagaa
GluA1 (as)	cgtcgctgacaatctcaagt
GluA2 (s)	gaccagaacggaaaacgaat
GluA2 (as)	ttcaagcccagatgtgtcat
GluA3 (s)	cctcctgatcctcccaatg
GluA3 (as)	cgctctctatgggggacacc
GluA4 (s)	agaaggacccagtgaccaac
GluA4 (as)	atgcagccagattagcagtg
GluA4 (s) (3′ end)	tgtagcgacgcccaaggg
GluA4 (as) (3′ end)	gtacggccttggggcagt
GluN1 (s)	gctgtacctgctggaccgct
GluN1 (as)	gcagtgtaggaagccacgatgatc
GluN2A (s)	gctacgggcagacagagaag
GluN2A (as)	gtggttgtcatctggctcac
GluN2A (s) (3′ end)	acagcaagaggagcaaatctc
GluN2A (as) (3′ end)	tgtacacacgtctattgctgc
GluN2B (s)	gctacaacacccacgagaagag
GluN2B (as)	gagagggtccacactttcc
GluN2C (s)	aaccacaccttcagcagcg
GluN2C (as)	gacttcttgcccttggtgag
GluN2D (s)	cgatggcgtctggaatgg
GluN2D (as)	agatgaaaactgtgacggcg
GluK1 (s)	gcccctctcaccatcacgtat
GluK1 (as)	tggtcgatagagccttgggca
GluK2 (s)	ttcctgaatcctctctccct
GluK2 (as)	caccaaatgcctcccactatc
GluK3 (s)	gggtgtcagctgtgtcctct
GluK3 (as)	gacagagctttgggcatcagt
GluK3 (s) (3′ end)	atcgccattctgcagctaca
GluK3 (as) (3′ end)	ataggaggctggggcttgtg
GluK4 (s)	caaaggcctgggaatggagaata
GluK4 (as)	ccgccgcctgggatggata
GluK5 (s)	cgacaccaagggctacggcat
GluK5 (as)	ccgccacgaagacagcaatga
synapsin-1 (s)	accctgggtgtttgcccagatg
synapsin-1 (as)	acccacaacttgtacctgtcagacat
synaptobrevin-2 (s)	ggtggatgaggtggtggacatc
synaptobrevin-2 (as)	gctgaagtaaacgatgatgatgatgagg
synaptophysin (s)	tggacgtggtgaatcagctggtg
synaptophysin (as)	aaagtacacttggtgcagcctgaatg
neuroligin-1 (s)	gatggaccagcgagaacattg
neuroligin-1 (as)	atcgatcacaggtccaaagg
PSD-95 (s)	ttgcagatcggagacaagat
PSD-95 (as)	gatctcattgtccaggtgct
β-actin (s)	cgttgacatccgtaaagacct
β-actin (as)	caaagccatgccaatgttgtctct

3 ′ end primers pick up the 3 ′ coding region of the corresponding gene (see also Figure 9). s = sense; as = antisense.

### Immunocytochemistry

After removal of the culture medium, adherent cells were briefly washed twice with PBS/A (PBS + 0.1% (w/v) BSA). The cells were fixed with 4% (w/v) PFA for 10 min at room temperature (RT) and incubated with the primary antibodies diluted in PBT1 (PBS + 1.0% (w/v) BSA + 0.1% (v/v) Triton X-100) at RT for 30 min. The following primary antibodies were used: anti-Oct4 (1:500; Santa Cruz Biotechnology), anti-nestin (1:500; rat401, Millipore), and anti-Pax6 (1:50; DSHB). After incubation with the primary antibody, the incubation with either Cy2- or Cy3-coupled species-specific secondary antibodies (1:500; Dianova) diluted in PBS/A was performed at RT for 30 min. Finally, the cells were washed twice with PBS and mounted in PBS/glycerol (2:1). Pictures were taken at an Axioplan2 microscope with the AxioCam HRc camera using the AxioVision 4.4 and 4.5 software (Zeiss).

### Western blotting

Total membrane fractions of tissues and cell cultures were obtained by hypotonic lysis (10 mM HEPES/KOH, 1.5 mM KCl, 10 mM MgCl_2_, 0.5 mM DTT) followed by ultracentrifugation to pellet membrane-bound proteins (100,000 g; 1 h). For SDS-PAGE, 50 μg of membrane proteins were loaded per sample. After transferring the proteins to nitrocellulose membranes, the membranes were reversibly stained with Ponceau S (0.2% Ponceau S, 3% trichloroacetic acid, 3% sulfosalicylic acid) to check for the proper transfer of proteins in each lane. For immunoblot analysis, the following primary antibodies were used: anti-GluA1 (1:1000; kind gift of Dr. Richard Huganir), anti-GluA2 (1:500; BD Biosciences), anti-GluA2/3 (1:500; Millipore), anti-GluN1 (1:1000; kind gift of Dr. Nils Brose), anti-GluN2A (1:1000; Millipore), anti-GluN2B (1:500; BD Biosciences), anti-GluK2/3 (1:500; kind gift of Dr. Robert Wenthold), anti-GluK5 (1:500; Tocris), anti-synaptophysin (1:10,000; Novus Biologicals), anti-PSD-95 (1:250; BD Biosciences), and anti-calnexin (1:500; Santa Cruz Biotechnology). For detection, appropriate HRP-conjugated secondary antibodies (1:10,000; Sigma) were used. Whole brain proteins of neonatal (P3) C57BL/6 mice served as positive control. For the removal of antibodies from previously probed Western blots to allow reprobing of the membranes with another antibody, the membranes were incubated for 2 h in stripping buffer (25 mM glycine; pH 2.0, 1% SDS).

### Patch-clamp recordings

Whole-cell recordings from ESCs were performed at −60 to −70 mV 1 day after plating, using an EPC-9 amplifier controlled by Pulse software (HEKA Elektronik). Currents were digitized at 10 kHz and Bessel-filtered at 3 kHz. Pipettes were pulled from borosilicate glass to resistances of 4–5 MΩ. The extracellular solution contained 140 mM NaCl, 4 mM KCl, 2 mM CaCl_2_, 1 mM MgCl_2_, and 10 mM HEPES adjusted to pH 7.3 with NaOH. The pipette solution contained 130 mM CsF, 2 mM MgCl_2_, 1 mM CaCl_2_, 11 mM EGTA, and 10 mM HEPES adjusted to pH 7.3 with KOH. Agonists (1–10 mM glutamate or 0.6–5 mM kainate) were prepared in extracellular solution and rapidly applied using a theta capillary mounted onto a piezo-driven actuator (PI Physik Instrumente) as described previously (Korber et al., 2007).

### Data analysis

qRT-PCRs results were analyzed using Roche LightCycler Software 3.5. Quantitative real time data was obtained by mathematical modeling (Pfaffl, 2001). The expression of the genes of interest was normalized to the expression of the housekeeping gene β-actin (2^ΔCt^ method). To additionally compare the expression of genes in the different stem cell types to their expression in a control, the data was also normalized to the expression of genes in mouse whole brain (2^ΔΔCt^ method). Data are shown as mean ± SE of the mean (s.e.m.). Statistics were calculated using Prism 5.0 software (GraphPad). To compare the expression of a given receptor subunit across various stem cell types, One-Way ANOVA followed by Tukey's multiple comparison *post-hoc* test was used. To compare the expression of a given receptor subunit across two different stem cell types, unpaired Student's *t*-test was used.

## Results

### Embryonic stem cells express iGluR mRNAs

**Figure 1:**
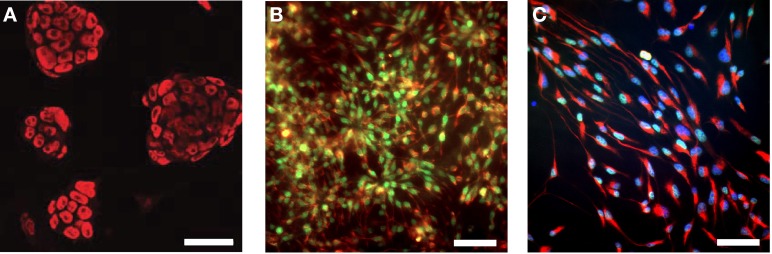
**Expression of embryonic and neural stem cell markers in 46C ESCs, NEPs, and NSCs. (A)** ESCs express the embryonic stem cell marker Oct4 (red fluorescence). **(B)** NEPs express the neuroepithelial marker Sox1 (green fluorescence), as well as the neural stem cell marker nestin (red fluorescence). **(C)** NSCs express the neural stem cell markers Pax6 (green fluorescence) and nestin (red fluorescence). Here, nuclei were stained with Hoechst 33342 (blue fluorescence). Scale bars: 50 μm **(A)** or 25 μm **(B,C)**.

In this study, we used the well-studied genetically engineered ESC line 46C, which expresses EGFP under the control of the early neuroectodermal marker Sox1. Thus, the generation of Sox1-positive NEPs from ESCs can be monitored via the expression of EGFP (Ying et al., 2003; Conti et al., 2005). Upon prolonged cultivation in a neuroinductive medium, NEPs differentiate into radial glia-like NSCs. Previous studies investigated the expression of a diverse panel of embryonic and NSC markers at the mRNA and protein level in the 46C ESCs, NEPs, and NSCs (Ying et al., 2003; Conti et al., 2005; Muth-Kohne et al., 2010a,b). In this study, we initially confirmed the protein expression of a representative set of stem cell markers in the investigated cell types (Figure 1). Undifferentiated 46C ESCs express the ESC marker Oct4 (Figure 1A; red fluorescence). Via cultivation in a neuroinductive medium, ESCs differentiate into NEPs. 46C NEPs are Sox1-positive, as indicated by their green fluorescence (Figure 1B). Additionally, NEPs express the NSC marker nestin (Figure 1B; red fluorescence). After differentiation into NSCs, the expression of Sox1 is downregulated and NSCs were shown to express the neural stem markers Pax6 (Figure 1C; green fluorescence) and nestin (Figure 1C; red fluorescence).

**Figure 2:**
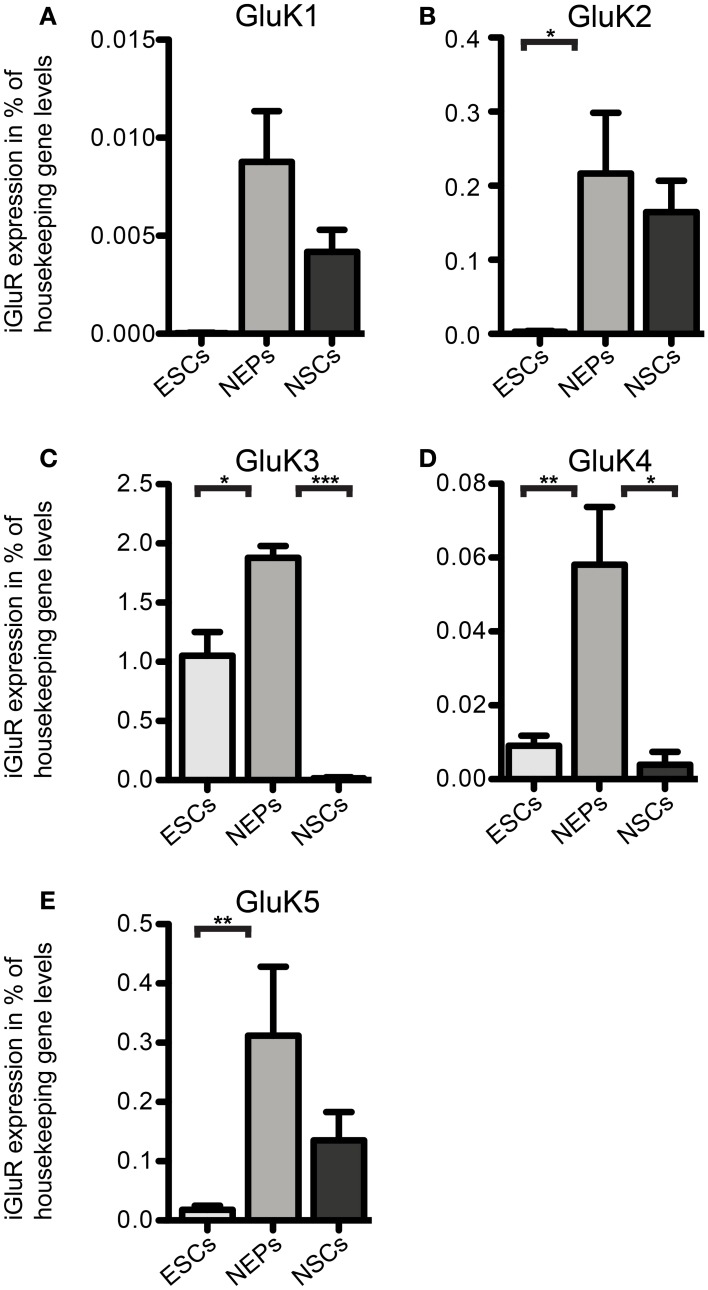
**Expression of KAR subunit mRNAs in 46C ESCs, NEPs, and NSCs normalized to the expression of the housekeeping gene β-actin (2^ΔCt^). (A)** GluK1. **(B)** GluK2. **(C)** GluK3. **(D)** GluK4. **(E)** GluK5. ESCs express KAR subunits at the RNA level; the most abundantly expressed KAR subunit in ESCs and NEPs is GluK3. The expression of all KAR subunits is upregulated upon differentiation into NEPs and subsequently downregulated in NSCs. Data represent mean ± s.e.m.; statistical significances were assigned by One-Way ANOVA followed by Tukey's multiple comparison *post-hoc* test; ^*^p < 0.05; ^**^p < 0.01; ^***^p < 0.001; *n* = 3–11 independent experiments. Note that statistical differences in expression of receptor subunits were only calculated between ESCs/NEPs, and NEPs/NSCs, respectively, not between ESCs/NSCs, since ESCs cannot differentiate into NSCs without differentiating into NEPs first.

**Figure 3:**
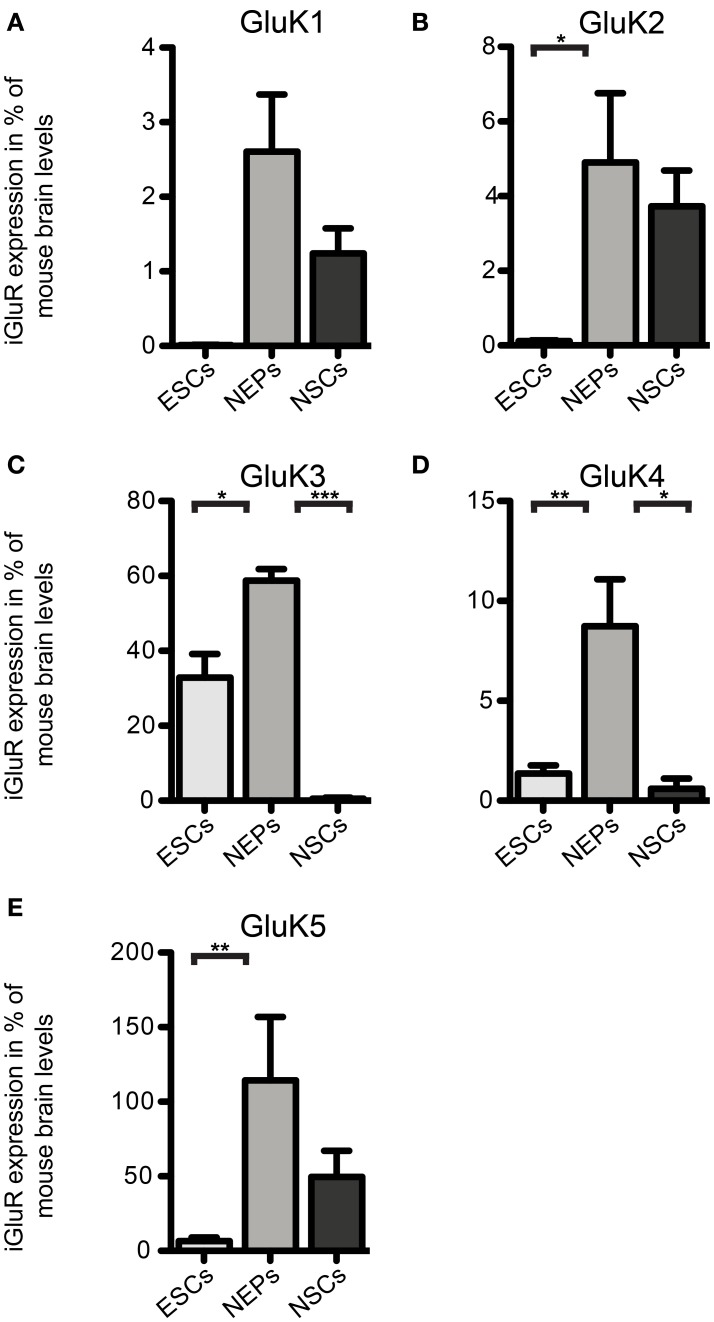
**Expression of KAR subunit mRNAs in 46C ESCs, NEPs, and NSCs normalized to the expression in whole mouse brain (2^ΔΔCt^). (A)** GluK1. **(B)** GluK2. **(C)** GluK3. **(D)** GluK4. **(E)** GluK5. Data represent mean ± s.e.m.; statistical significances were assigned by One-Way ANOVA followed by Tukey's multiple comparison *post-hoc* test; ^*^p < 0.05; ^**^p < 0.01; ^***^p < 0.001; *n* = 3–11 independent experiments. Note that statistical differences in expression of receptor subunits were only calculated between ESCs/NEPs, and NEPs/NSCs, respectively, not between ESCs/NSCs, since ESCs cannot differentiate into NSCs without differentiating into NEPs first.

**Figure 4:**
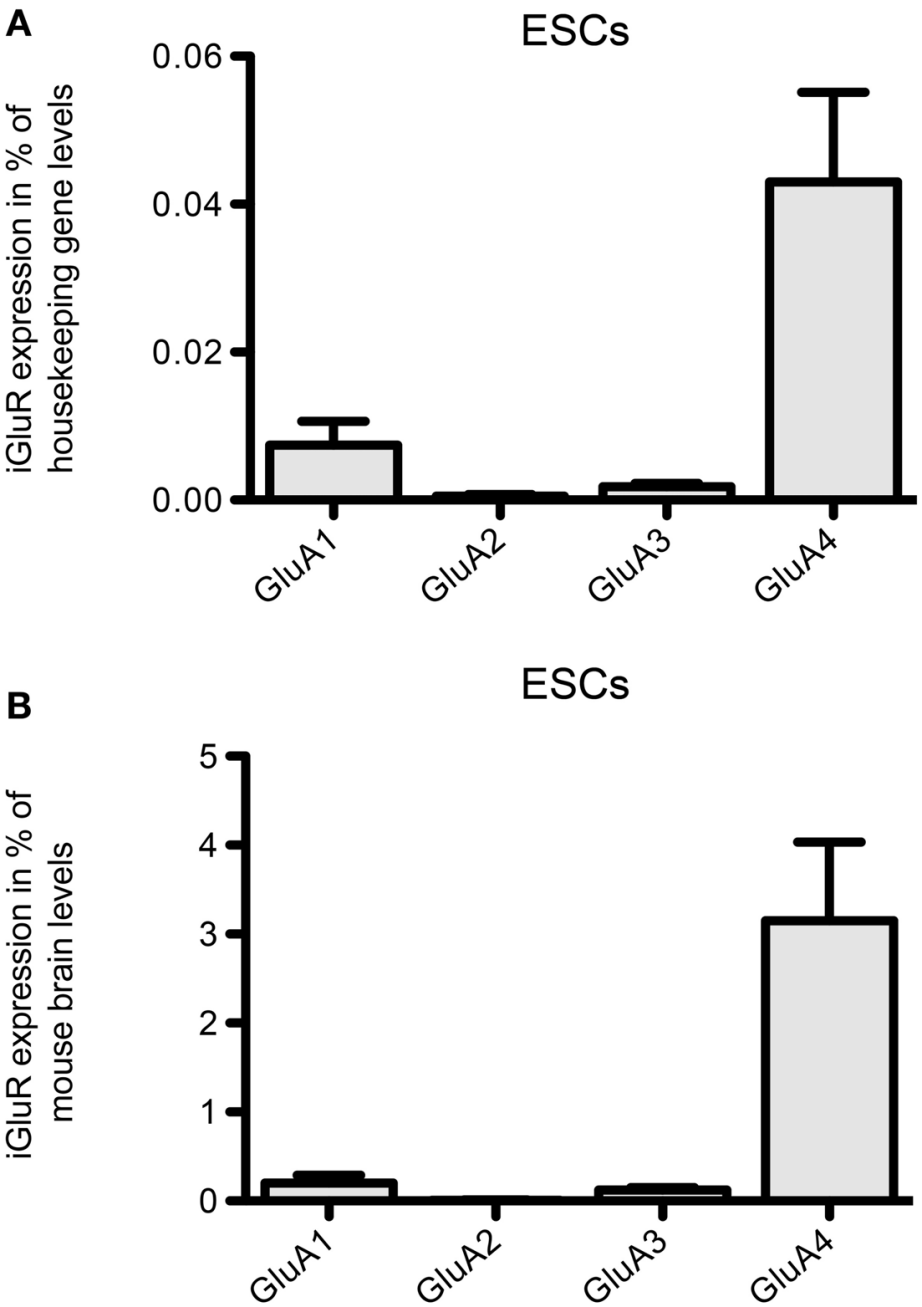
**Expression of AMPAR subunit mRNAs in 46C ESCs. (A)** Expression normalized to the expression of the housekeeping gene β-actin (2^ΔCt^). **(B)** Expression normalized to the expression in whole mouse brain (2^ΔΔCt^). Whereas GluA2 is not expressed in ESCs, and GluA1 and GluA3 are only weakly expressed, ESCs robustly express the AMPAR subunit GluA4. Data represent mean ± s.e.m.; *n* = 6–10 independent experiments. Note that, here, no statistical differences were calculated, since we did not compare the expression of different receptor subunits within one cell type, but only across different cell types.

**Figure 5:**
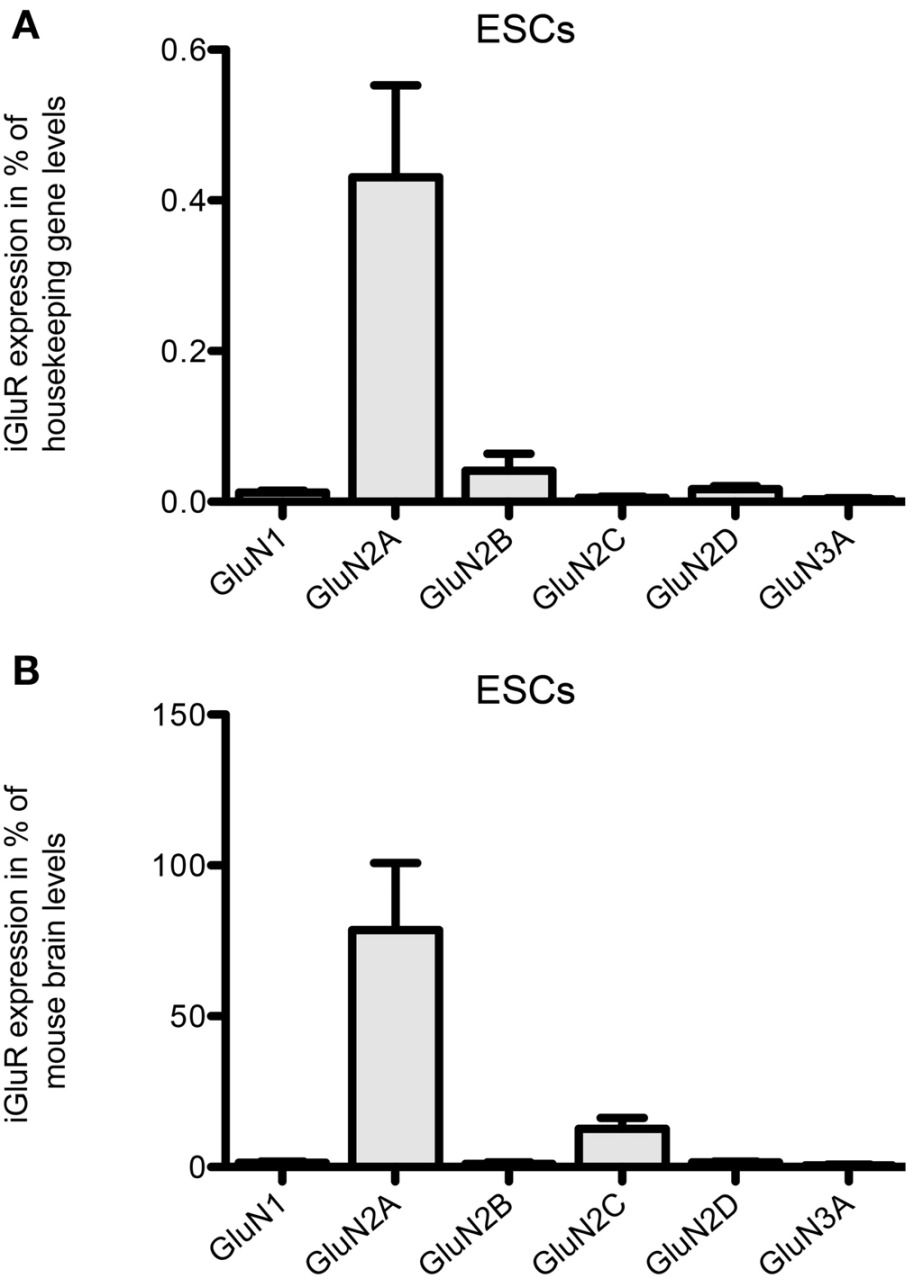
**Expression of NMDAR subunit mRNAs in 46C ESCs. (A)** Expression normalized to the expression of the housekeeping gene β-actin (2^ΔCt^). **(B)** Expression normalized to the expression in whole mouse brain (2^ΔΔCt^). The obligatory NMDAR subunit GluN1 is only weakly expressed in ESCs; the most abundantly expressed NMDAR subunit in ESCs is GluN2A. Data represent mean ± s.e.m.; *n* = 4–5 independent experiments. Note that, here, no statistical differences were calculated, since we did not compare the expression of different receptor subunits within one cell type, but only across different cell types.

Next, we investigated the expression of iGluR mRNAs in undifferentiated 46C ESCs via qRT-PCR. Surprisingly, we found mRNA of all three subfamilies to be expressed in ESCs, albeit most of them were only weakly expressed and some subunits barely reached detection threshold (Figures 2–5). Among the probed iGluRs, two subunits are particularly highly expressed in 46C ESCs, namely the KAR subunit GluK3 and the NMDAR subunit GluN2A. Besides GluK3, 46C ESCs weakly express the KAR subunits GluK2, GluK4, and GluK5 (Figures 2, 3).

We also investigated the mRNA expression profile of KARs in cells of later developmental stages (46C NEPs and NSCs). In contrast to ESCs, NEPs and NSCs weakly express GluK1 (Figure 2). Regarding NEPs, the KAR subunits GluK2 to GluK5 are significantly upregulated in comparison to their expression in ESCs (Figures 2, 3). The strongest expressed KAR subunits in NEPs are GluK3 and GluK5 (Figure 2).

By comparing the expression of KAR subunits in NSCs to their expression in NEPs, we found all KAR subunits to be downregulated in NSCs (Figures 2, 3). In particular, the expression of GluK3 and GluK4 is significantly decreased in NSCs. Similar to NEPs, the KAR subunit GluK5 is strongly expressed in NSCs, as is GluK2. During the time course of development, we thus find an upregulation in the mRNA expression of GluK2 and GluK5, which are the major KAR subunits found at mature hippocampal mossy fiber synapses (Mulle et al., 1998; Contractor et al., 2003) and are generally regarded as the prevalent KAR heteromers in the adult brain (Lerma, 2003).

Regarding the mRNA expression of AMPAR subunits in 46C ESCs, the expression of GluA3 barely reached detection threshold and GluA1 is only weakly expressed (Figure 4). In contrast to that, 46C ESCs robustly express GluA4, since the expression of GluA4 in 46C ESCs accounts for ~3% compared to its expression in mouse whole brain (Figure 4B).

Moreover, NMDAR transcripts are expressed in 46C ESCs (Figure 5). In particular, we found a strong mRNA expression of GluN2A in undifferentiated 46C ESCs, which equals ~80% of its expression in mouse whole brain (Figure 5B). Previously, we already have analyzed NMDA and AMPA receptor expression at later developmental cell stages (Muth-Kohne et al., 2010a,b); whereas GluN2A remains the only robustly expressed NMDAR subunit also in 46C NEPs and NSCs, the AMPAR subunits GluA2, GluA3, and GluA4 are strongly upregulated in 46C NEPs and NSCs (Muth-Kohne et al., 2010a,b).

### Embryonic stem cells do not express functional iGluR protein complexes

**Figure 6:**
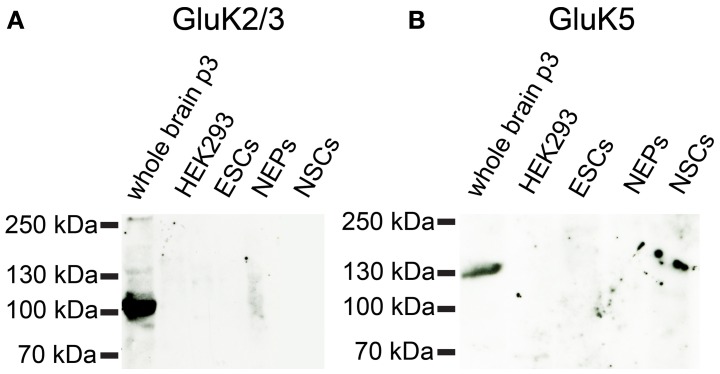
**Expression of KAR subunit proteins in 46C ESCs, NEPS, and NSCs**. Protein isolated from neonatal whole mouse brain (P3) was used as a positive control; protein from HEK293 cells served as negative control. **(A)** GluK2/3. **(B)** GluK5. A band at the expected molecular weight of the receptor subunits (115 kDa for GluK2/3 and 120 kDa for GluK5) is only visible in the positive control. Neither ESCs nor NEPs or NSCs express GluK2/3 or GluK5 at the protein level.

Based on the mRNA profiling results, we raised the question whether 46C ESCs not only express iGluR mRNAs, but also the corresponding protein complexes. Therefore, we performed Western blot analyses of plasma membrane proteins. As the strongest expressed iGluRs in 46C ESCs are the KAR subunits GluK3 and GluK5 (see Embryonic stem cells express iGluR mRNAs), we first investigated their protein expression in 46C ESCs. Both primary antibodies were specific, since they yielded a strong signal at the expected molecular weight (~120 kDa) in the positive control (plasma membrane proteins isolated from P3 mouse whole brain), but not in the negative control (plasma membrane proteins isolated from HEK293 cells) (Figure 6). However, we could not detect any GluK2/3 or GluK5 protein expression in 46C ESCs. Moreover, also cells of later developmental stages (NEPs and NSCs) do not express GluK2/3 or GluK5 at the protein level (Figure 6), although the mRNA expression of KAR subunits is strongly upregulated in NEPs (see Embryonic stem cells express iGluR mRNAs).

**Figure 7:**
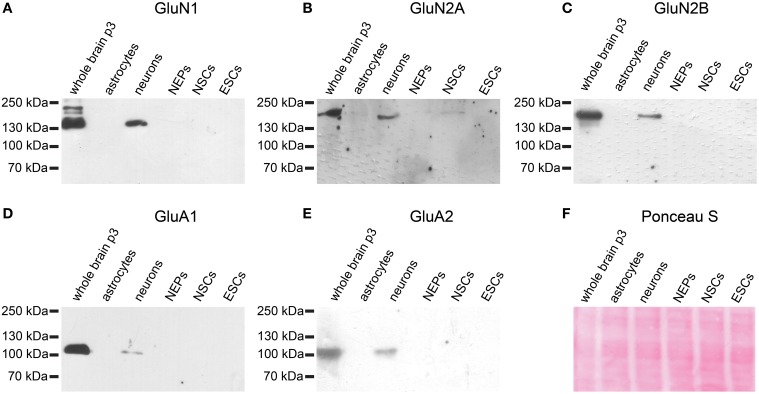
**Expression of AMPAR and NMDAR subunit proteins in 46C ESCs, NEPs, and NSCs**. Protein isolated from neonatal whole mouse brain (P3) was used as a positive control. Additionally, protein from early neurons and astrocytes was applied. **(A)** GluN1. **(B)** GluN2A. **(C)** GluN2B. **(D)** GluA1. **(E)** GluA2. All antibodies yielded a band at the expected molecular weight (130 kDa for GluN1, 170 kDa for GluN2A and GluN2B, 105 kDa for GluA1, and 100 kDa for GluA2) in the positive control. ESCs do not express any of the investigated NMDAR or AMPAR subunit proteins. **(F)** Exemplary Ponceau S staining of proteins blotted on a nitrocellulose membrane.

Similarly, neither the NMDAR subunits GluN1, GluN2A, or GluN2B nor the AMPAR subunits GluA1 or GluA2 are expressed at the protein level in 46C ESCs (Figure 7). By contrast, early neurons, which were differentiated from 46C NEPs via retinoic acid treatment, express both AMPAR and NMDAR proteins (Figure 7). Moreover, 46C NSCs weakly express GluN2A at the protein level, as we have shown previously (Muth-Kohne et al., 2010b).

**Figure 8:**
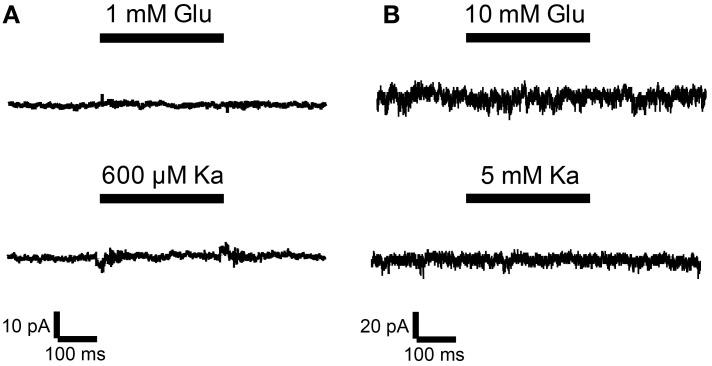
**Whole-cell patch-clamp recordings were performed on 46C ESCs**. ESCs did not show any currents upon application of agonists at either low concentrations [**(A)**; 1 mM glutamate or 0.6 mM kainate; *n* = 14] or high agonist concentrations [**(B)**; 10 mM glutamate or 5 mM kainate; *n* = 45].

Thus, undifferentiated 46C ESCs do express iGluR mRNAs, but they do not express iGluR proteins. To finally rule out the possibility of functional iGluR complexes in 46C ESCs, we performed whole-cell patch-clamp recordings of 46C ESCs. However, no current responses were detectable upon rapid direct application of either the endogenous agonist glutamate or the partial agonist kainate (Figure 8). This holds true both for low (1 mM glutamate or 0.6 mM kainate, Figure 8A) and high (10 mM glutamate or 5 mM kainate, Figure 8B) agonist concentrations. These results clearly show that 46C ESCs do not express functional iGluR complexes at the plasma membrane.

**Figure 9:**
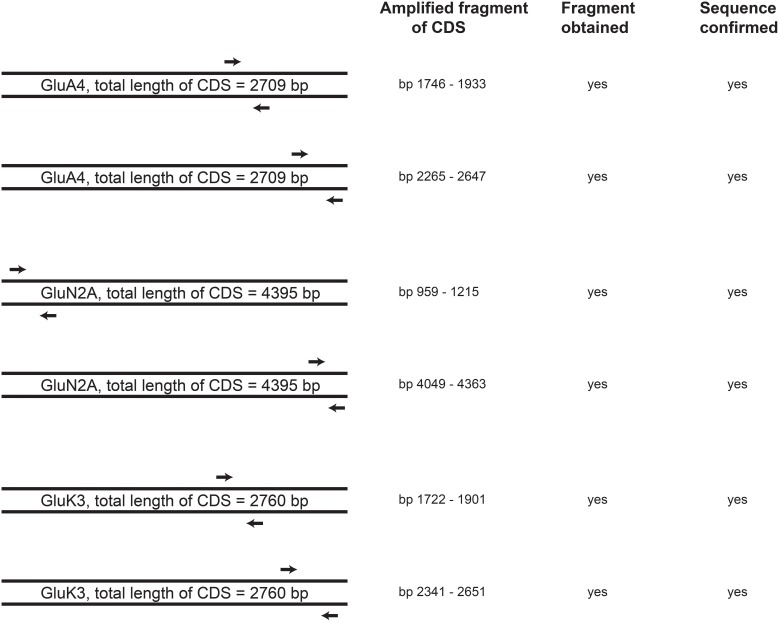
**Schematic drawing of the position of primers in the CDS of the highest expressed receptor subunits in 46C ESCs (GluA4, GluN2A, and GluK3)**. 3′ end primers (second primer pair for each receptor subunit), which pick up the 3′ region of the CDS, were used in qRT-PCRs to verify the full-length expression of receptor transcripts in undifferentiated 46C ESCs. For all investigated iGluR subunits, fragments of the appropiate size were obtained and verified by DNA sequencing. Thus, full-length expression of GluA4, GluN2A, and GluK3 transcripts in undifferentiated 46C ESCs was confirmed, *n* = 3 independent experiments.

To investigate whether 46C ESCs express full-length transcripts of iGluRs or whether only truncated transcripts are expressed, we used an additional, different set of primers for the highest expressed receptor subunits of each iGluR family in 46C ESCs (namely GluA4, GluN2A, and GluK3). These primers pick up the 3′ coding region of the corresponding gene (Figure 9). Following qRT-PCRs, the amplified fragments were sequenced using an ABI 3130xl capillary sequencer (Applied Biosystems). GluA4, GluN2A, and GluK3 are indeed expressed as full-length transcripts in undifferentiated 46C ESCs, as confirmed by DNA sequencing.

**Figure 10:**
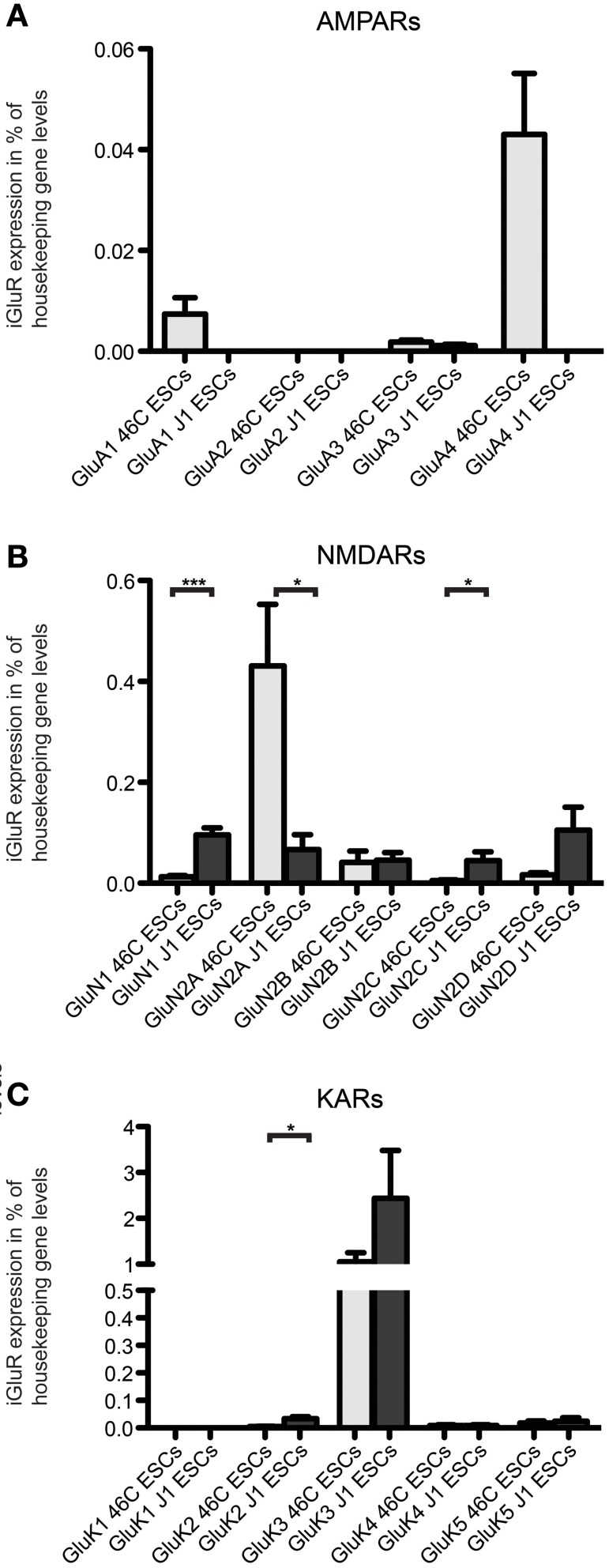
**Expression of AMPAR (A), NMDAR (B), and KAR (C) mRNAs in 46C ESCs and J1 ESCs normalized to the expression of the housekeeping gene β-actin (2^ΔCt^)**. Undifferentiated J1 ESCs express iGluR subunits at the RNA level. The only expressed AMPAR subunit in J1 ESCs is GluA3, which is only weakly expressed. In contrast to that, J1 ESCs express transcripts of all NMDAR subunits, and the expression of GluN1 and GluN2C is significantly higher in J1 ESCs than in 46C ESCs. The strongest expressed KAR transcript in both 46C ESCs and J1 ESCs is GluK3. Data represent mean ± s.e.m.; statistical significances were assigned by unpaired Student's *t*-test; ^*^p < 0.05; ^***^p < 0.001; *n* = 3–11 independent experiments.

Additionally, we checked the expression of iGluR transcripts in a different, non-engineered ESC line (J1 ESCs). We found transcripts of all three iGluR families to be expressed in undifferentiated J1 ESCs (Figure 10), albeit their expression does not exactly match the expression of receptor subunits in undifferentiated 46C ESCs.

The only expressed AMPAR subunit in J1 ESCs is GluA3, which is only weakly expressed in these cells. GluA1, GluA2, and GluA4 are not expressed in J1 ESCs (Figure 10A). In contrast to that, 46C ESCs do not only express GluA3, but also GluA1 and, most prominently, GluA4 (Figures 4, 10A). Regarding NMDAR subunits, J1 express all NMDAR subunits at the RNA level (Figure 10B). In comparison to 46C ESCs, the expression of GluN1 and GluN2C is significantly higher in J1 ESCs than in 46C ESCs. Conversely, GluN2A is significantly lower expressed in J1 ESCs than in 46C ESCs. The mRNA expression of KARs in J1 ESCs is similar to their expression in 46C ESCs: GluK1 is neither expressed in J1 ESCs nor in 46C ESCs, and GluK2, GluK4, and GluK5 are only weakly expressed at the RNA level in both ESC lines. GluK3 is the highest expressed KAR subunit in both J1 and 46C ESCs, and its expression does not differ significantly between both ESC types (Figure 10C).

**Figure 11:**
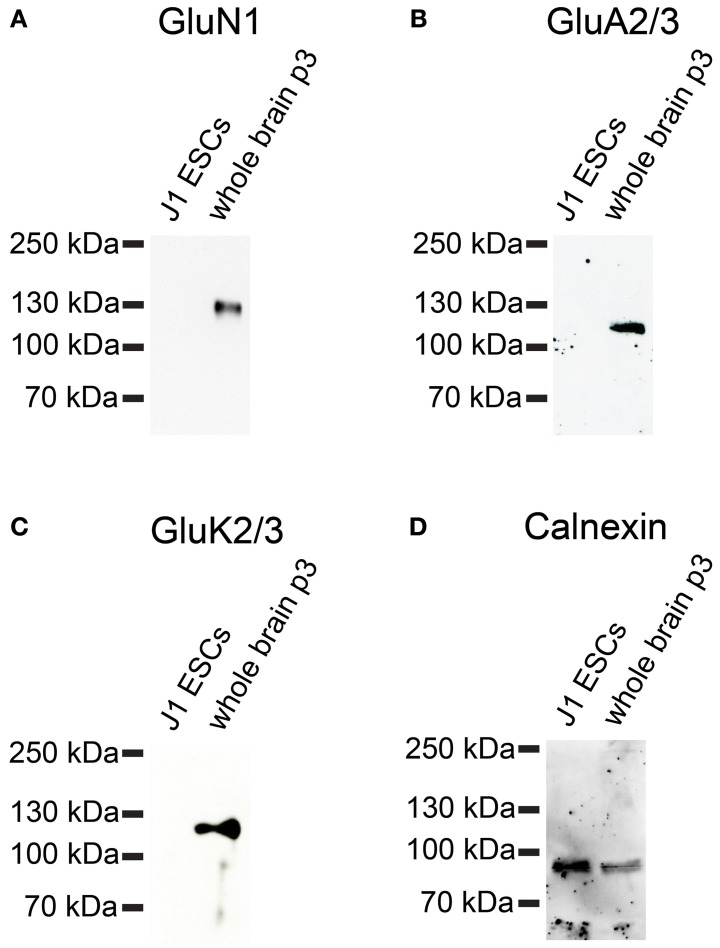
**Expression of iGluR subunit proteins in J1 ESCs**. Protein isolated from mouse whole brain (P3) served as positive control. **(A)** GluN1. **(B)** GluA2/3. **(C)** GluK2/3. A band at the expected molecular weight of the receptor subunits (130 kDa for GluN1, 110 kDa for GluA2/3, and 115 kDa for GluK2/3) is only visible in the positive control. J1 ESCs do not express any of the investigated iGluR subunits at the protein level. **(D)** Expression of the housekeeping protein calnexin (90 kDa). High background staining results from the fact that this blot had previously been probed with another antibody and had been stripped before re-probing.

Next, we investigated whether iGluR subunits are expressed at the protein level in undifferentiated J1 ESCs. Therefore, we performed Western blots with plasma membrane proteins isolated from J1 ESCs and used antibodies directed against GluN1, GluA2/3, and GluK2/3 to probe these blots. J1 ESCs do not express any of the investigated iGluR subunits at the protein level (Figure 11), confirming that lack of receptor protein expression in 46C ESCs (Figures 6, 7) in presence of respective mRNA expression is not a unique property of 46C ESCs.

### Stem cells express glutamatergic synapse marker mRNAs, but not the corresponding proteins

After establishing the expression of iGluR mRNAs but failing to detect any corresponding plasma membrane-resident protein complexes, we investigated whether 46C ESCs, NEPs, and NSCs also express the mRNAs of other proteins present at typical glutamatergic synapses. Therefore, we determined the mRNA expression of the presynaptically expressed synaptic vesicle proteins synapsin-1, synaptobrevin-2, and synaptophysin, as well as of the postsynaptic markers neuroligin-1 and PSD-95.

**Figure 12:**
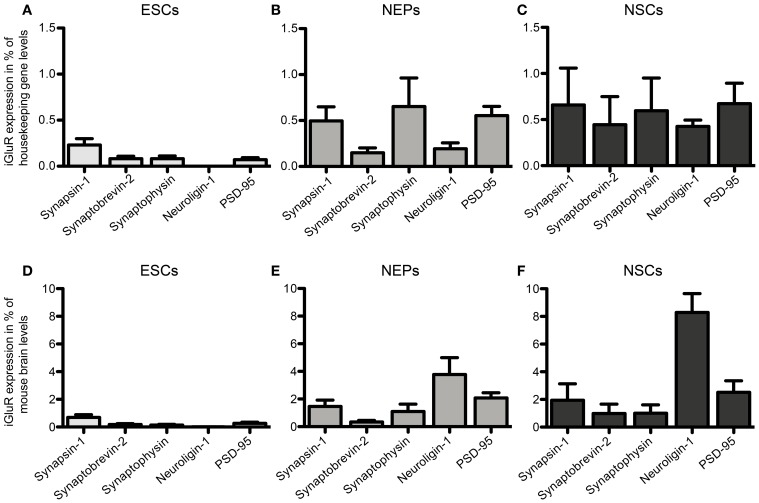
**Expression of presynaptic (synapsin-1, synaptobrevin-2, synaptophysin) and postsynaptic (neuroligin-1 and PSD-95) marker mRNAs in 46C ESCs, NEPs, and NSCs normalized to the expression of the housekeeping gene β-actin (A–C) (2^ΔCt^) and the expression in whole mouse brain (D–F) (2^ΔΔCt^). (A,D)** Expression in ESCs. **(B,E)** Expression in NEPs. **(C,F)** Expression in NSCs. ESCs weakly express pre- and postsynaptic markers at the mRNA level. The expression of synaptic markers is then continuously upregulated upon differentiation into NEPs and NSCs. Data represent mean ± s.e.m.; *n* = 5–6 independent experiments. Note that statistical differences in the expression of markers were only calculated between ESCs/NEPs, and NEPs/NSCs, respectively, not between ESCs/NSCs, since ESCs cannot differentiate into NSCs without differentiating into NEPs first. Between ESCs/NEPs and NEPs/NSCs, no significant differences were found by One-Way ANOVA followed by Tukey's multiple comparison *post-hoc* test.

**Figure 13:**
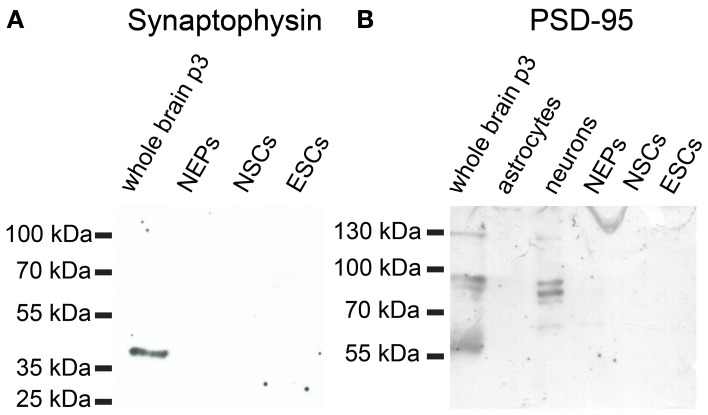
**Expression of synaptic marker proteins in 46C ESCs, NEPs, and NSCs**. Protein isolated from early neurons and/or neonatal whole mouse brain (P3) was used as a positive control. **(A)** Synaptophysin (38 kDa). **(B)** PSD-95 (95 kDa). Neither type of stem cell expresses synaptophysin or PSD-95 proteins.

46C ESCs robustly express mRNAs of the presynaptic marker synapsin-1, whereas synaptobrevin-2, synaptophysin, and PSD-95 are only weakly expressed (Figure 12). mRNA for the postsynaptic marker neuroligin-1 could not be detected at all in ESCs. During the development from ESCs to NEPs and, subsequently, to NSCs, all synaptic marker mRNAs are upregulated. However, while the mRNA levels of synapsin-1 and synaptophysin are comparable between 46C NEPs and NSCs, the mRNAs of synaptobrevin-2, neuroligin-1, and PSD-95 are upregulated in NSCs (Figure 12), although this upregulation is not statistically significant. However, as for the iGluR subunits, we were unable to confirm the presence of the presynaptic marker synaptophysin or of the postsynaptic marker PSD-95 at the protein level in 46C ESCs (Figure 13). A band at the expected molecular weight (38 kDa for synaptophysin and 95 kDa for PSD-95) is only visible in early neurons differentiated from 46C NEPs or in membrane proteins isolated from whole mouse brain (Figure 13). Regarding PSD-95, there is an additional band visible (~60 kDa) in early neurons, and a very faint crossreactive band in NEPs, which might depict a cross-reacting protein. However, in NEPs, no band is visible at the expected molecular weight of PSD-95.

## Discussion

### Undifferentiated embryonic stem cells express iGluR transcripts

In the present study, we analyzed the expression of KARs, AMPARs, and NMDARs in 46C and J1 ESCs as well as the expression of KARs in 46C-derived NEPs and NSCs. Surprisingly, we found subunits of all three iGluR families to be expressed at the mRNA level in both ESC lines, although we could not detect any iGluR protein expression. Additionally, we ruled out the possibility of functional iGluRs in undifferentiated 46C ESCs, as whole-cell patch-clamp recordings gave no currents upon glutamate or kainate application. We also investigated the expression of several glutamatergic synapse marker mRNAs in 46C ESCs, NEPs, and NSCs, and found that all three cell types express both pre- and postsynaptic markers at the RNA level. However, similar to our findings regarding iGluR expression, the mRNA expression of synaptic markers in stem cells does not result in protein expression.

Recent studies indicate that a promiscuous transcriptional activity, which does not necessarily result in protein translation, might be a general principle of undifferentiated ESCs (Sperger et al., 2003; Golan-Mashiach et al., 2005; Meshorer and Misteli, 2006; Efroni et al., 2008). This holds also true for lineage- and tissue-specific genes (Efroni et al., 2008). The unique structure of chromatin in ESCs might be the reason for their global transcriptional activity, since their chromatin is generally maintained in an open conformation (Meshorer and Misteli, 2006). Furthermore, the chromatin of ESCs shows a looser binding of architectural proteins (Meshorer and Misteli, 2006). Taken together, these features of ESCs might explain the mRNA expression of iGluRs and glutamatergic synapse markers, although their transcription does not lead to protein translation.

So far, only few studies analyzed the expression of KARs during rodent brain development, in which the mRNA of KAR subunits was detected as early as E10 in rat embryos (Bahn et al., 1994; Scherer and Gallo, 1998; Lilliu et al., 2002; Ritter et al., 2002). We now raised the question whether KAR transcripts are expressed in undifferentiated ESCs as well and found that mRNAs for all KAR subunits except for GluK1 are expressed in undifferentiated 46C and J1 ESCs, with GluK3 mRNA being the most abundant. After differentiation from 46C ESCs to NEPs, the expression of all KAR mRNAs is significantly increased and, subsequently, decreased when NEPs are differentiated into NSCs (Figures 2, 3).

To further investigate the expression of KAR subunits in 46C ESCs, NEPs, and NSCs, we performed Western blot analyses with GluK2/3 and GluK5 antibodies. We found that neither GluK2, GluK3, nor GluK5, are expressed at the protein level in any of the analyzed cell types (Figure 6). Additionally, undifferentiated J1 ESCs do not express GluK2 or GluK3 subunits at the protein level either (Figure 11).

Besides the expression of KARs, we also investigated the expression of AMPARs and NMDARs in undifferentiated ESCs. Mirroring our findings for KAR expression in ESCs, 46C and J1 ESCs express AMPAR and NMDAR subunits at the RNA level, but these transcripts are not translated into receptor proteins.

Regarding AMPARs, it has been shown that their expression starts early during development (Monyer et al., 1991; Jansson et al., 2011). We now found that undifferentiated 46C ESCs also express GluA1 and GluA4 transcripts (Figure 4), and that in cells of later developmental stages (i.e., NEPs and NSCs) the expression of GluA2-GluA4 is upregulated (Muth-Kohne et al., 2010a,b).

Interestingly, 46C ESCs robustly express GluA4 mRNA. In contrast to that, the only expressed AMPAR subunit in undifferentiated J1 ESCs is GluA3. Thus, GluA4 is not expressed at all in J1 ESCs. This difference in AMPAR expression at the RNA level in 46C and J1 ESCs might be due to the fact that 46C and J1 cells are different ES cell lines of different origin with a different culturing history.

Nevertheless, we could not detect any AMPAR protein expression neither in 46C ESCs, NEPs, or NSCs, nor in J1 ESCs (Figures 7, 11).

Similar to AMPARs and KARs, NMDARs are present early during neural development (Monyer et al., 1994; Liu et al., 2004). We found that although undifferentiated 46C ESCs express also GluN2B transcripts, the most strongly expressed NMDAR subunit at the mRNA level in 46C ESCs is GluN2A (Figure 5). Nevertheless, we found weak protein expression of GluN2A only in 46C NSCs (Figure 7) (Muth-Kohne et al., 2010b). However, the obligatory NMDAR subunit GluN1 is not present at the protein level, neither in 46C ESCs, NEPs, nor NSCs, although it is expressed at the mRNA level (Figures 5, 7) (Muth-Kohne et al., 2010a,b). Thus, functional NMDARs cannot assemble in any of these three cell types. Similar to that, undifferentiated J1 ESCs express transcripts of all NMDAR subunits (Figure 10), and the expression of GluN1 is even significantly higher in J1 ESCs than in 46 ESCs. Nevertheless, GluN1 is not expressed at the protein level in J1 ESCs either (Figure 11).

Additionally, we confirmed the presence of full-length transcripts of the strongest expressed receptor subunits (namely GluA4, GluN2A, and GluK3) in undifferentiated 46C ESCs (Figure 9). Thus, full-length transcripts of iGluRs are present in undifferentiated 46C ESCs, but they are not translated into receptor proteins.

### Undifferentiated embryonic stem cells do not form functional iGluRs

In addition to the analysis of iGluR mRNA expression patterns, we also investigated the expression of pre- and postsynaptic markers in 46C ESCs, NEPs, and NSCs. In general, the mRNA expression of all synaptic markers examined in this study is slightly upregulated during differentiation from ESCs to NEPs and NSCs (Figure 12).

However, synaptophysin is not expressed at the protein level, neither in 46C ESCs, NEPs, nor NSCs (Figure 13). Similar to that, the staining of synapsin-1 and synaptophysin proteins is restricted to cells with neuronal morphology when ESCs are differentiated into neuronal cells by treatment with retinoic acid (Finley et al., 1996).

Regarding the postsynaptic marker PSD-95, we could not detect its protein expression in any of the investigated stem cell types (Figure 13), but only in whole mouse brain and neurons differentiated from 46C NEPs. Nevertheless, there is a very faint crossreactive band visible in NEPs at a lower molecular weight (~60 kDa) than the expected molecular weight of PSD-95 (95 kDa). This might depict an unrelated cross-reacting protein.

Among the presynaptic markers investigated in this study, synapsin-1 is robustly expressed at the mRNA level in undifferentiated 46C ESCs, as well as in NEPs and NSCs. Synapsin-1 has been shown to be expressed early (E12–14) during development and to play a key role in the maturation of glutamatergic synapses and in the biogenesis of synaptic vesicles (Melloni and Degennaro, 1994; Zurmohle et al., 1994; Bogen et al., 2009; Vasileva et al., 2012).

Thus, similar to the expression of iGluR transcripts, which are not translated into receptor proteins, undifferentiated ESCs do express pre- and postsynaptic markers at the mRNA level, but not at the protein level.

It has been demonstrated that neurons derived from ESCs via treatment with retinoic acid show currents in response to the application of NMDA or kainate (Bain et al., 1995; Finley et al., 1996; Kim et al., 2009). In the present study, we raised the question whether undifferentiated ESCs could express functional iGluRs as well. First, we observed robust mRNA expression of the KAR subunits GluK3 and GluK5 as well as the AMPAR subunit GluA4 and the NMDAR subunit GluN2A in undifferentiated 46C ESCs. However, we could not detect any protein expression of iGluRs in ESCs. To rule out the possibility of an insufficient sensitivity of the protein detection method, we performed patch-clamp recordings of 46C ESCs. Undifferentiated 46C ESCs did not show any current responses when glutamate or kainate were applied to the cells (Figure 8). This is in line with the observed lack of receptor proteins in undifferentiated ESCs. Similar to our results, Maric et al. (2000) showed that neural precursors from E13 rat neuroepithelium express iGluRs at the mRNA level, while glutamate-activated currents could only be observed in differentiating neurons, not in proliferating neural precursors (Maric et al., 2000).

In summary, undifferentiated 46C and J1 ESCs were shown to express subunits of all three major subfamilies of iGluRs (KARs, AMPARs, and NMDARs) at the mRNA level. Similar to that, pre- and postsynaptic marker mRNAs were present in 46C ESCs and in cells of later developmental stages, namely NEPs and NSCs. However, with the exception of GluN2A in NSCs, none of these transcripts could be detected at the protein level. Additionally, the expression of functional iGluRs in 46C ESCs was ruled out since patch-clamp recordings of 46C ESCs gave no currents upon iGluR agonist application. It has been postulated before that ESCs globally express a wide variety of genes, including lineage- and tissue-specific transcripts. Our study shows that mRNAs of all three major iGluR subfamilies are expressed in ESCs as well, although their transcription does not result in protein translation or the formation of functional iGluRs.

One possible explanation for this phenomenon might be a microRNA-mediated inhibition of iGluR subunit translation. It is now well known that microRNAs play important roles during development and differentiation (Rosa and Brivanlou, 2009, 2013). Although most of the studies focus on microRNAs that influence and regulate the self-renewal and pluripotency of stem cells (Houbaviy et al., 2003; Gangaraju and Lin, 2009; Wang et al., 2009), one cannot rule out the possibility of microRNAs that target iGluRs in ESCs. It has been shown previously that microRNA-132 plays a critical role during the differentiation of dopaminergic neurons from ESCs (Yang et al., 2012). Interestingly, microRNA-132 also regulates the expression of GluN2A, GluN2B, and GluA1 through a BDNF-dependent mechanism (Kawashima et al., 2010). Thus, it will be an interesting question for future studies if microRNA-132 or other microRNAs are also involved in the translational regulation of iGluRs in stem cells and if those miRNAs are expressed in 46C ESCs as well.

### Conflict of interest statement

The authors declare that the research was conducted in the absence of any commercial or financial relationships that could be construed as a potential conflict of interest.
